# Enantioselective dearomatization reactions of heteroarenes by anion-binding organocatalysis

**DOI:** 10.1039/d2cc07101k

**Published:** 2023-02-15

**Authors:** Martin Aleksiev, Olga García Mancheño

**Affiliations:** a Organic Chemistry Institute, University of Münster, Corrensstraße 36/40 48149 Münster Germany olga.garcia@uni-muenster.de

## Abstract

Catalytic asymmetric dearomatization of heteroaromatic compounds has received considerable attention in the last few years, since it allows for a fast expansion of the chemical space by converting relatively simple, flat molecules into complex, three dimensional structures with added value. Among different approaches, remarkable progress has been recently achieved by the development of organocatalytic dearomatization methods. In particular, the anion-binding catalysis technology has emerged as a potent alternative to metal catalysis, which together with the design of novel, tunable anion-receptor motifs, has provided new entries for the enantioselective dearomatization of heteroarenes through a chiral contact ion pair formation by activation of the electrophilic reaction partner. In this feature, we provide an overview of the different methodologies and advances in anion-binding catalyzed dearomatization reactions of different heteroarenes.

## Abstract 2

This feature article outlines the up-to-date implementation of anion-binding catalysis for the enantioselective dearomatization of hereoarenes, expanding the chemical space by converting available flat building blocks into valuable chiral molecules.

## Graphical abstract

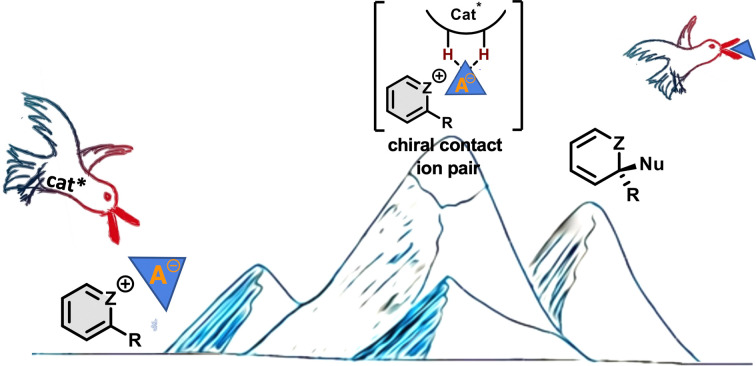

## 1. Introduction

Aromatic compounds are broadly distributed in nature and excellent substrates of choice for synthesis,^1^ since there are plentiful commercially and readily available derivatives of generally low cost. The dearomatization of these compounds is very useful for expanding the chemical space by converting relatively simple, flat molecules into more decorated, complex, three dimensional structures with added value for several research fields such as organic synthesis, agrochemical and pharmaceutical chemistry.^2–6^ Despite the synthetic advantages of this straightforward strategy, the high stability of the aromatic compounds makes the control of the regioselectivity and stereoselectivity of this process very challenging.^7^ However, in the last decade, a progress in asymmetric catalytic dearomati-zation reactions has shown the great potential of this area.^8–10^

**Fig. 1:**
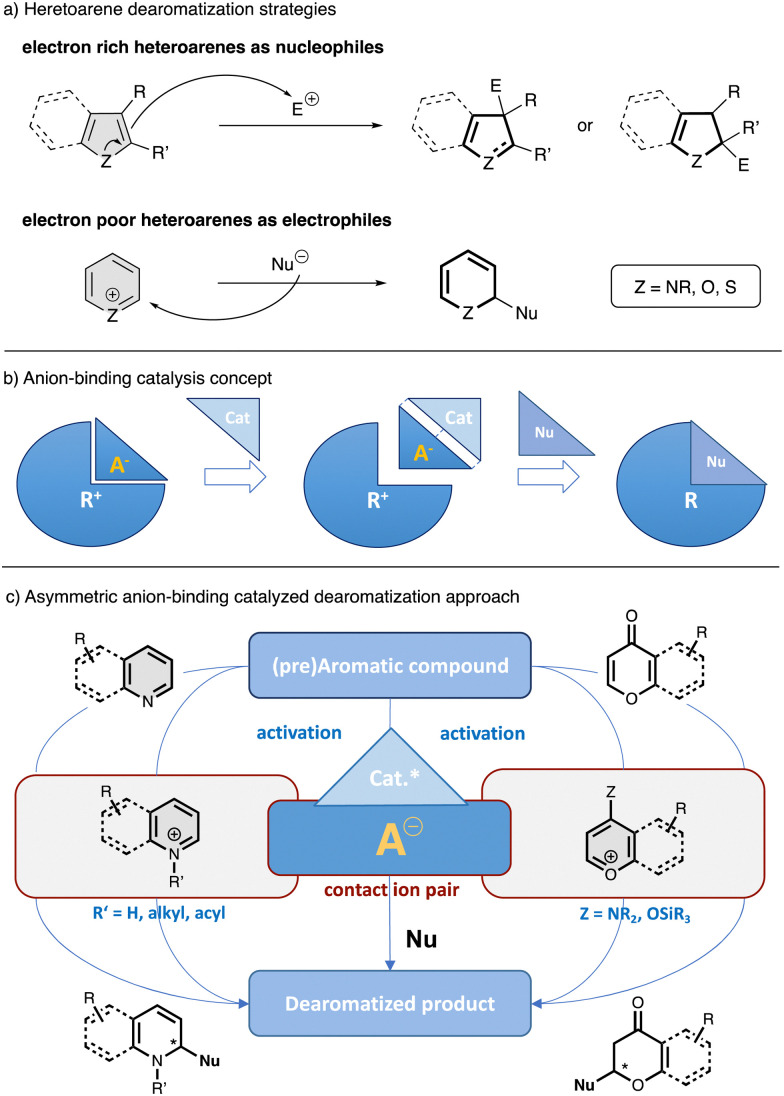
General scheme for the dearomatization of heteroarenes (a) anion-binding concept (b) and asymmetric anion-binding dearomatization approach *via* activation-nucleophilic addition (c).

The enantioselective dearomatization of heteroarenes is of particular interest due to the fact that heterocycles are key elements of natural products and pharmaceuticals.^11–13^ Catalytic asymmetric dearomatization reactions of heteroarenes could be carried out by different methodologies, in which the arene can be enrolled as nucleophile or electrophile depending of its intrinsic nature (Fig. 1a).^8,14^ In this regard, transition metal asymmetric catalysis has been extensively used in this type of processes due to the extended possibilities provided by organometallic chemistry and the availability of versatile chiral ligands.^8,15–17^ Nevertheless, most of these methods are still limited regarding to the dearomatization of electron poor arenes, such as pyridines or (iso)quinolines, as they are generally more efficient for electron rich systems. In contrast, the development of several organocatalytic methods has allowed an efficient activation of electrophilic heteroarenes and the introduction of chirality by dearomatization through ionic intermediates.^8,18,19^ Due to the fact that most organic reactions proceed *via* charged species or the presence of some degree of charge polarization, finding efficient ways to control the stereochemistry with this kind of species lies at the heart of organic synthesis. Among the different catalytic approaches involving charged intermediates or reagents, anion-binding has recently emerged as a powerful toolkit in asymmetric catalysis.^19–26^ Anion-binding catalysis^19^ is based on the activation of an ionic electrophile by the binding of a catalyst to its counter anion through non-covalent interactions such as hydrogen-bonding, forming a chiral contact ion-pair complex, which can induce chirality into the final product after a nucleophilic attack (Fig. 1b). It is worthy to mentioned that, in the last decades, a large number of small molecules have been described to operate through an anion-binding activation mechanism.^19^ Most of these catalysts rely on hydrogen bonding,^27,28^ but present additional structural elements to help for the substrate activation and stabilization of the transition state by different contacts such as additional hydrogen bonds or π–π interactions.^19–31^ Some of the most prominent catalysts that operate through this mechanism and have been employed for the development of enantioselective dearomatization of heteroarenes are chiral thioureas, silenodienols and oligo-triazoles, among others.

In the field of anion-binding catalyzed dearomatization reactions, typically the *in situ* generation of the reactive ionic electrophilic heteroarene is required (Fig. 1c).^19^ Thus, a previous activation of the heteroarene or pre-aromatic precursor by nitrogen atom quaternization though protonation, alkylation or acylation (N-heteroarenes), or by enamine formation or enolization (O-heteroarenes), allows the following enantioselective nucleophilic addition in the presence of a chiral catalyst. Additionally, there is also possible the dearomatization of electron rich hetereoarenes, such as indoles, by facilitating their stereocontrolled attack to anion-binding activated electrophilic species.

Several reviews on the broad use of anion-binding in catalysis,^19–26^ as well as asymmetric dearomatization reactions of arenes^8–10^ have been published. However, this feature just focusses on the different methodologies and advances in enantioselective anion-binding catalyzed strategies. We aim at giving an overview of the dearomatization of different types of heteroarenes embracing this activation mode, in which our group has contributed significantly in the past few years. In this feature, the examples reported are arranged according to the type of aromatic substrate, and it has been further divided by the nature of the nucleophile and/or catalyst employed in each reaction.

## 2. Dearomatization of isoquinolines

### 2.1. Silyl ketene acetals as nucleophiles

**Scheme 1:**
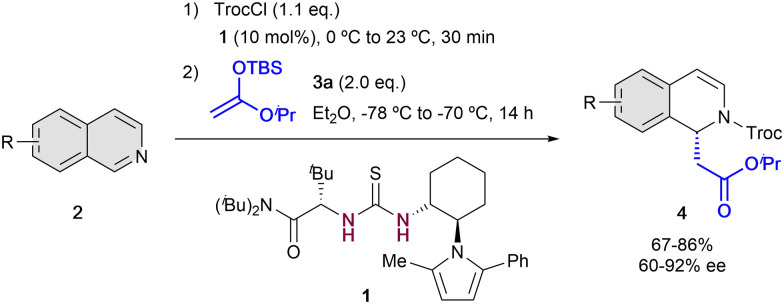
First anion binding dearomatization reaction reported by Jacobsen *et al*.

The first example of enantioselective anion-binding catalyzed dearomatization of an N-heteroarene was published in 2005 by Jacobsen and co-workers (Scheme 1).^32^ It consists in an *N*-acyl Mannich reaction of isoquinolines 2 employing 10 mol% of the chiral thiourea catalyst 1 and *tert*-butyldimethylsilyl (TBS) protected ketene acetal 3a as a nucleophile. In this case, strong dependence in the solvent and the acylating group was found. The best results were obtained with non-polar solvents as diethyl ether and 2,2,2-trichloroethyl chloroformate (TrocCl) as acylating agent. As in Reissert-type reactions,^33,34^ the activation of the substrate by forming the *N*-acyl iminium cation was first performed, followed by the formation of the chiral contact ion pair in presence of the chiral organocatalyst. Lastly, the nucleophilic addition takes place towards the corresponding 1,2-dihydroquinolines 4, which were obtained with good yields and high enantioselectivities up to 92% ee.

**Scheme 2:**
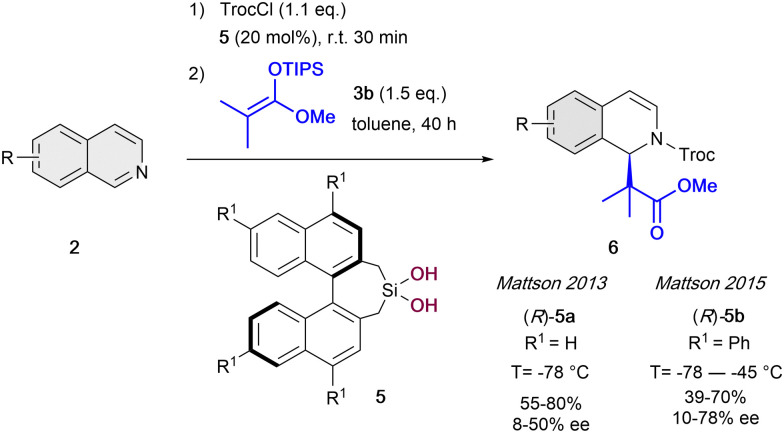
Silanediol-catalyzed dearomatization of isoquinolines by Mattson *et al*.

After this pioneering work, in the following years other several catalytic systems have been introduced as alternative anion-binding organocatalysts. In 2013, the Mattson group developed the first example of a C2-symmetric 1,1′-bi-2-naphtol (BINOL) based silanediol HB-donor 5 as anion-binding catalyst (Scheme 2).^35^ They were able to achieve moderated yields and enantioselectivities up to 50% ee in the presence of 20 mol% of (*R*)-5a. The best results were obtained employing TrocCl, and triisopropylsilyl (TIPS) dimethyl ketene acetal 3b in toluene at −78 °C. Motivated by the initial success, in 2015 they further improved the levels of enantiocontrol to up to 78% ee by employing the re-designed tetraphenyl-substituted silanediol catalyst (*R*)-5b at −55 °C.^36^

Regarding to the hardly explored silanediol catalysts, it can be remarked the later development of biphenyl-2,2-bisfenchol-based (BIFOL) silanediol by Goldfuss *et al.* in 2019.^37^ This alkoxysilanediol has an unexpectedly stable BIFOL backbone against hydrolysis, and is able to achieve the dearomatization of isoquinolines by *N*-acyl Mannich reaction in high yields, though only in low enantioselectivities (up to 12% ee).

**Scheme 3:**
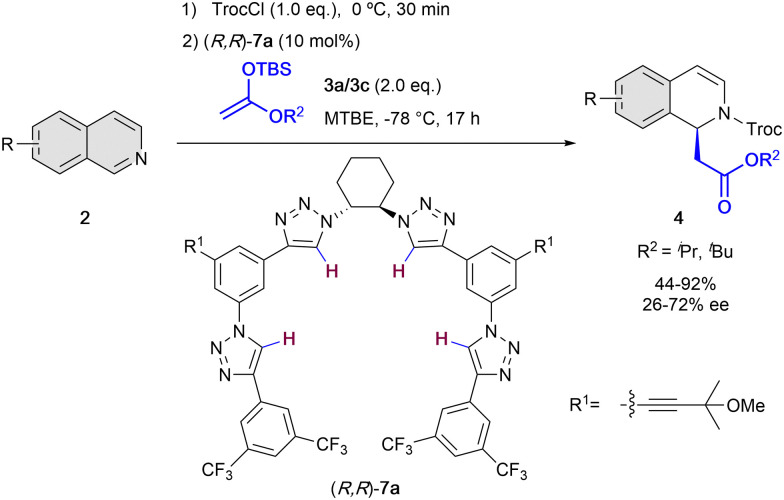
Chiral oligotriazole-catalyzed dearomatization of isoquinolines by García Mancheño *et al*.

On the other hand, in 2014, the García Mancheño group developed a new family of helical triazole-based HB-donors 7 as anion-binding catalysts.^38^ 1,2,3-Triazoles are heterocycles with interesting properties such as large dipole moment (*μ* = 4.38 D)^39^ and highly polarizable C–H bonds,^40^ what makes them promising HB-donor moieties for anion-binding catalysis.^41^ The chirality in the triazole catalysts 7 is introduced by a chiral *trans*-1,2-diamine unit that acts as a central backbone to pre-orientate the formation of the chiral helical structure upon binding to the substrate counter-anion. Nevertheless, this central chirality is not enough to induce high level chirality transfer, given that four triazole rings are required to have a cooperative effect towards the effective formation of a chiral helical triazole-anion complex.^38^ In 2016, our group employed the triazole organocatalysts 7 for the *N*-acyl Mannich reaction of isoquinolines 2 with silyl ketene acetals 3 (Scheme 3).^42^ The reaction was carried out in presence of 10 mol% of (*R*,*R*)-7a, TrocCl as acylating agent, and methyl *tert*-butyl ether (MTBE) as solvent at −78 °C, achieving high yields and enantioselectivities up to 72% ee.

### 2.2. Extension to other nucleophiles with thiourea catalysts

**Scheme 4:**
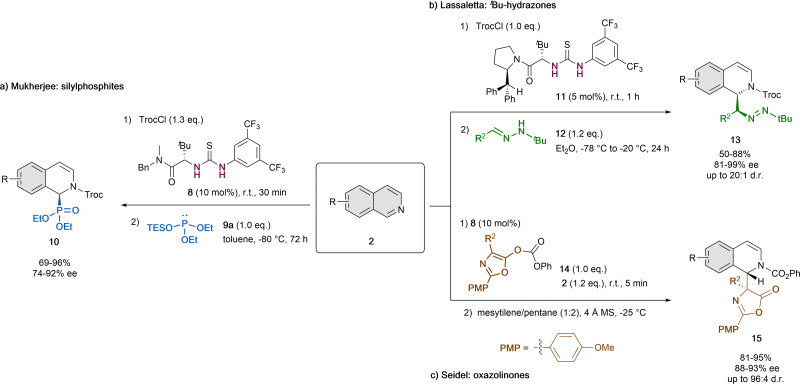
Thiourea-catalyzed asymmetric dearomatization of isoquinolines by (a) Choudhury and Mukherjee, (b) Lassaletta *et al.* and (c) Seidel *et al*.

In addition to the pioneering work of Jacobsen (Scheme 1),^32^ in the last years thiourea organocatalysts were also used together with different nucleophiles for the reaction with the same type of *in situ* formed *N*-acyl isoquinolinium cation by acylation of isoquinolines with TrocCl (Scheme 4).

In 2016, Choudhury and Mukherjee reported the asymmetric dearomatization of isoquinolines 2 employing 10 mol% of the *tert*-leucine-based thiourea derivative 8 and diethyl triethylsilyl (TES) phosphite 9a as a nucleophile in toluene at −80 °C (Scheme 4a).^43^ As a result, the corresponding cyclic α-aminophosphonates 10 were generated with high yields and enantioselectivities up to 92% ee. This method provides an easy way to obtain chiral α-aminophosphonates, that are widely used in medicinal and pharmaceutical science.^44^

Alternatively, non-silylated nucleophiles were also applied. In 2021, Lassaletta and co-workers described the ability of *tert*-leucine-derived thiourea 11 to catalyze the dearomatization reaction of isoquinolines 2 with *N-tert*-butyl hydrazones 12 as polarity-reversed nucleophiles in Et_2_O (Scheme 4b).^45^ The resulting dihydro-isoquinolines 13 present two contiguous stereogenic centers and were obtained in high yields, diastereoselectivities up to 20 : 1 d.r. and enantioselectivities up to 99% ee.

Moreover, azalactones (3-oxazolin-5-ones) could also be enrolled in this type of catalytic dearomatization reactions *via* a Steglich rearrangement, which was reported by Seidel *et al.* in 2011 (Scheme 4c).^46^ To this end, they used 10 mol% of the thiourea organocatalyst 8, 4 Å molecular sieves and a mixture of mesitylene and pentane (1 : 2) as solvent at −25 °C. Upon prior formation of the *N*-acylisoquinolinium ion by acylation from the azalactone 14, the attack of the enolate species evolves to the final disubstituted amino acid products 15 in high yields, enantioselectivities up to 93% ee and diastereoselectivities up to 96 : 4 d.r.

## 3. Dearomatization of quinolines

### 3.1. Strategies based on hydrogen-bonding catalysis

Compared to isoquinolines, quinolines proved to be more challenging substrates, most probably because of the lower reactivity and inefficient interaction to the mostly employed bidentate organocatalysts. In contrast, dual or multidentate systems are able to achieve better stereocontrol in the dearomatization process of quinolines through a better fixation of the *N*-acylquinolinium salt.

**Scheme 5:**
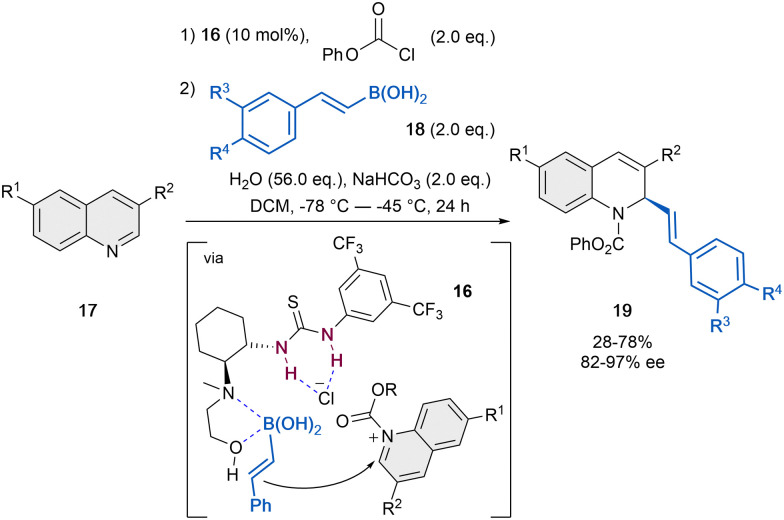
Asymmetric Petasis-type reaction of quinolones reported by Takemoto *et al*.^47^

The presumably first anion-binding catalytic dearomatization of quinolines 17 relied on a Petasis-type reaction reported by Takemoto *et al.* in 2007 (Scheme 5).^47^ The reaction was carried out by employing 10 mol% of the dual catalyst 16 in the presence of phenyl chloroformate as acylating agent, vinyl boronic acid (18) as a nucleophile, a mixture of H_2_O and NaHCO_3_ for the regeneration of the catalyst and CH_2_Cl_2_ as solvent. While the 1,2-amino alcohol group of the catalyst activates the boronic acid, the thiourea unit can bind the chloride counter-anion and form a contact ion pair with the *N*-phenoxycarbonyl quinolinium salt. Thus, the corresponding dihydroquinolines 19 were achieved in moderate yields and enantioselectivities up to 97% ee.

**Scheme 6:**
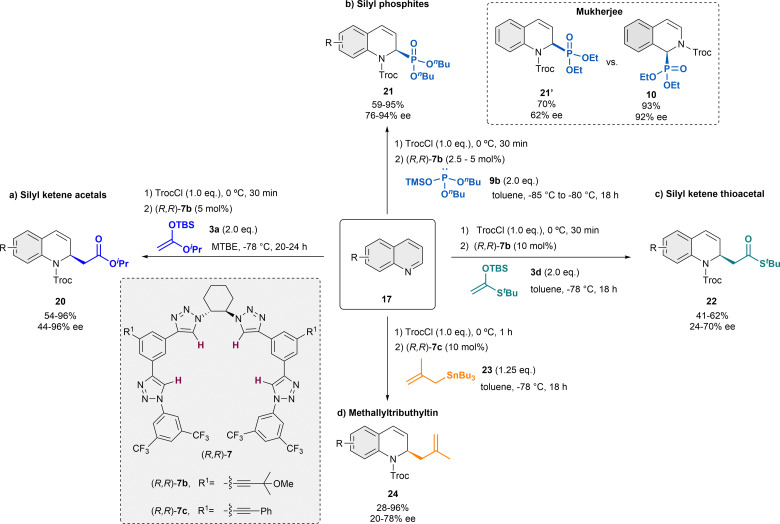
Asymmetric dearomatization of quinolines employing several nucleophiles in the presence of tetrakis-triazole catalysts 7.

In the following years, the García Mancheño group has made a significant contribution to the development of different catalytic systems for the dearomatization of quinolines, by *in situ* standard activation through acylation with TrocCl (Scheme 6).^38,48–52^ In 2014, we reported Reisert-type reaction of quinolines 17 employing 5 mol% of the regioisomer tetrakistriazole (*R*,*R*)-7b, in presence of iso-propyl ketene acetal 3a in MTBE as solvent (Scheme 6a).^38^ Electron rich and electron poor substituents were well tolerated and the desired products 20 were obtained with high yields and enantioselectivities up to 96% ee. Only substituents in C-8 were not tolerated, and substituents in C-3 hindered the correct evolution of the reaction, leading to significantly lower enantioselectivities.

The application of this catalytic system could be efficiently extended to different nucleophiles. Inspired by the thiourea-catalyzed studies of Mukherjee with phosphites, which were not performing that well with quinolines (*e.g.* 62% ee, quinoline *vs.* 92% ee, isoquinoline),^43^ the related asymmetric triazole-catalyzed dearomatization towards the synthesis of the corresponding dihydroquinoline α-aminophosphonic acid derivatives 21 was developed (Scheme 6b).^48^ In order to achieve the desired products, silyl protected alkylphosphites such as 9b [(^*n*^BuO)_2_P-OTMS] were used in presence of 2.5 or 5 mol% of (*R*,*R*)-7b in toluene. Both electron donating and withdrawing groups were well tolerated, obtaining the products 21 high yields and enantioselectivities up to 94% ee.

After the successful studies with silyl ketene acetals and phosphites presenting a similar nucleophilicity according to the Mayr's nucleophilicity scale^53,54^ (*N* ∼ 10), our group further screened several nucleophiles for this approach.^49^ The selected nucleophiles presented *N*-values between 1.7 and 10. It was observed that nucleophiles with *N*-value below 6 do not react under the standard conditions and strong nucleophiles, such as Grignard reagents with expected *N* > 20, aggressively reacted with the substrate leading to decomposition products. However, it was noticed that the yield and enantioselectivity could not be perfectly correlated to the *N*-value. The best chiral inductions were obtained with the related silyl ketene thioacetal 3d (Scheme 6c).^49^ Hence, after the corresponding optimization process, the desired dihydroquinolines 22 were obtained with moderate yields and enantioselectivities up to 72% ee, in presence of 10 mol% of (*R*,*R*)-7b in toluene.

Furthermore, methallyltributhyltin 23 (*N* = 7.5) also provided promising results during the nucleophile screening.^49^ Therefore, an asymmetric α-allylation of quinolines was developed embracing a similar strategy (Scheme 6d).^50,55^ This approach opens a fast access to chiral N-heterocycles bearing a versatile allyl group that could be transformed into further valuable groups. For this reaction, 10 mol% of catalyst (*R*,*R*)-7c bearing additional aromatic substituents in the backbone for a better interaction with the allyltin reagent was used in dry toluene. Hence, the α-allylated dihydroquinolines 24 were obtained in high yields and enantioselectivities up to 78% ee.

**Fig. 2:**
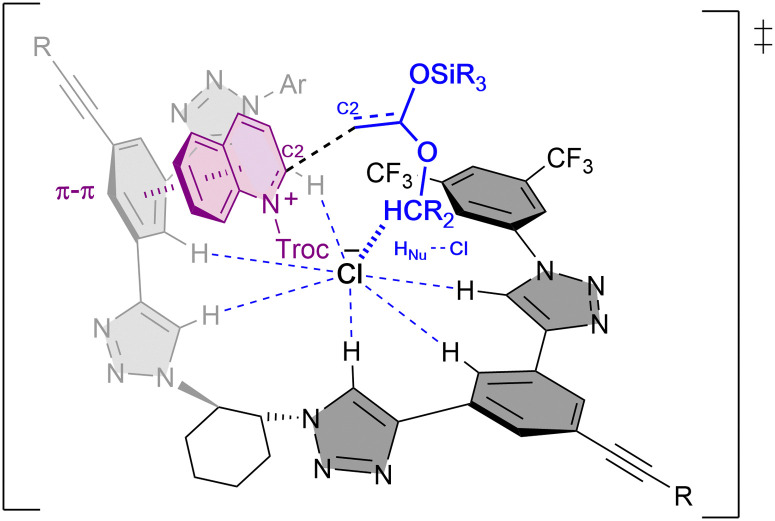
Plausible transition state and key interactions between (*R*,*R*)-7b and the quinolinium substrate in the chiral contact ion-pair complex based on experimental and computational studies.

Combined experimental and computational mechanistic studies were carried out to gain important information on the key interactions between the catalyst and heteroarene substrate (Fig. 2).^51^

These studies showed that the main interaction towards the catalyst-anion complex formation is produced between the chloride and the hydrogen atoms of the triazole rings of the catalyst (*R*,*R*)-7b (*e.g.* K_a_(Cl^−^) = 536 M^−1^ in acetone-d_6_, 2 mM; Bu_4_NCl as Cl^−^ source). Moreover, the chloride is also interacting through hydrogen bonding with the substrate and the nucleophile, such as a silyl ketene acetal, guiding the addition process. Lastly, a π–π interaction between the substrate and one of the arms of the catalyst leads to a parallel orientation, allowing only the attack from the less hindered Re-face. All together it explains the obtention of the (*R*)-enantiomer as major product.

**Scheme 7:**
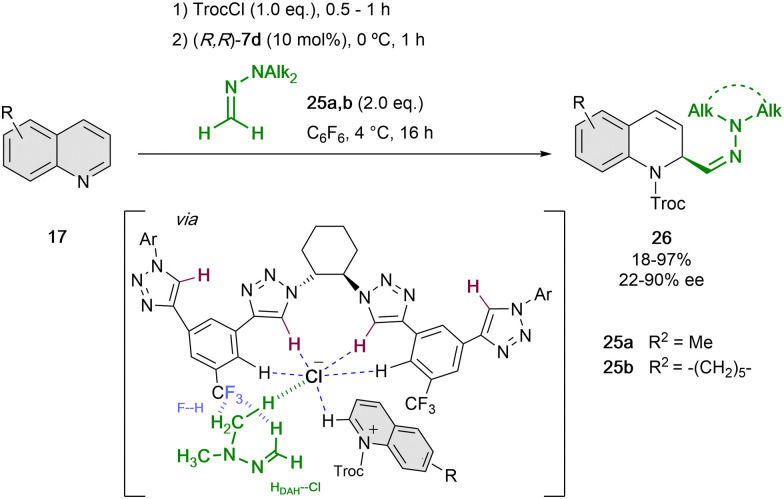
Enantioselective dearomatization of quinolines employing *N*,*N*-dialkylhydrazones reported by García Mancheño *et al*.

Based on these findings, we re-designed the triazole-based catalyst to (*R*,*R*)-7d, aiming at being able to enrol challenging formaldehyde *N*,*N*-dialkylhydrazones (DAHs)^56^ as polarity reverse nucleophiles (*N* ∼7–8) for the addition to the quinolinium salts (Scheme 7).^52^ Hence, in order to enhance the anion-binding affinity and allow new F–H bonds between the catalyst and the hydrogen atoms of the hydrazone, a CF_3_ group instead of the alkyne substituent was introduced in the side-arms of the tetrakistriazole catalyst. Moreover, the *N*,*N*-dialkyl substitution should guarantee for an additional hydrogen bond interaction with the counter-anion^57^ and a better fixation of both substrate and nucleophile in the catalyst cavity.

Computational studies showed that only one of the arms of the catalyst is interacting with the counterion, and the approach of the nucleophile is directed by the previously mentioned F–H bonds. For this reaction, 10 mol% of (*R*,*R*)-7d was used in C_6_F_6_, with the purpose to disfavour the possible cation–π interactions between the substrate and solvent.^58^ Different *N*,*N*-dialkylhydrazones 25 could be employed. However, the *N*-substitution affected significantly to the enantiocontrol, which is in concordance with the hypothesis of the participation of the *N*-alkyl group in the chiral contact ion pair.^57^ Different substitution of the quinoline was also well tolerated and the desired dihydroquinolines 26 were obtained in high yields and enantioselectivities up to 90% ee.

### 3.2. Strategies based on halogen-bonding catalysis

In the last years, halogen bonding (XB)^59,60^ has become a promising tool in organocatalysis,^61–63^ resembling and, in some cases, showing a stronger activation that the one exhibited by the more classical hydrogen bonding analogous interaction. The presence of an EWG attached to a halogen atom produces an anisotropically distributed electron density along the EWG–X bond, thus generating an electropositive region called σ-hole.^64^ This region, provides Lewis acidity properties to the halogen atoms, which allowed them to form non-covalent interactions with Lewis basic substrates.^59,60,65,66^ The strength of this interactions increases with the polarizability of the halogen. Consequently, most of the employed XB-organocatalysts are based on the iodine atom.^67^

Plausibly, the first reported reaction employing XB activation was published by Bolm and co-workers in 2008.^68^ In this work, a quinoline reduction was achieved in presence of a perfluoroalkene catalyst and a Hantzsch ester as a reducing agent. However, the first asymmetric version based primarily on halogen bonding activation was achieved by Huber *et al.* in 2020.^69^ They used a dicationic XB-donor catalyst based on two iodoimidazolium units with rigidly attached chiral sidearms for the asymmetric Mukaiyama aldol type reaction between an 1,2-dicarbonilic compound and a silyl-protected enolate. Unfortunately, only up to 33% ee could be attained, most probably due to the challenging nature of the XB-interaction that requires a high directionality (∼180°) and the larger distance between the substrate and the chiral backbone produced by the large size of the iodine atoms.

**Scheme 8:**
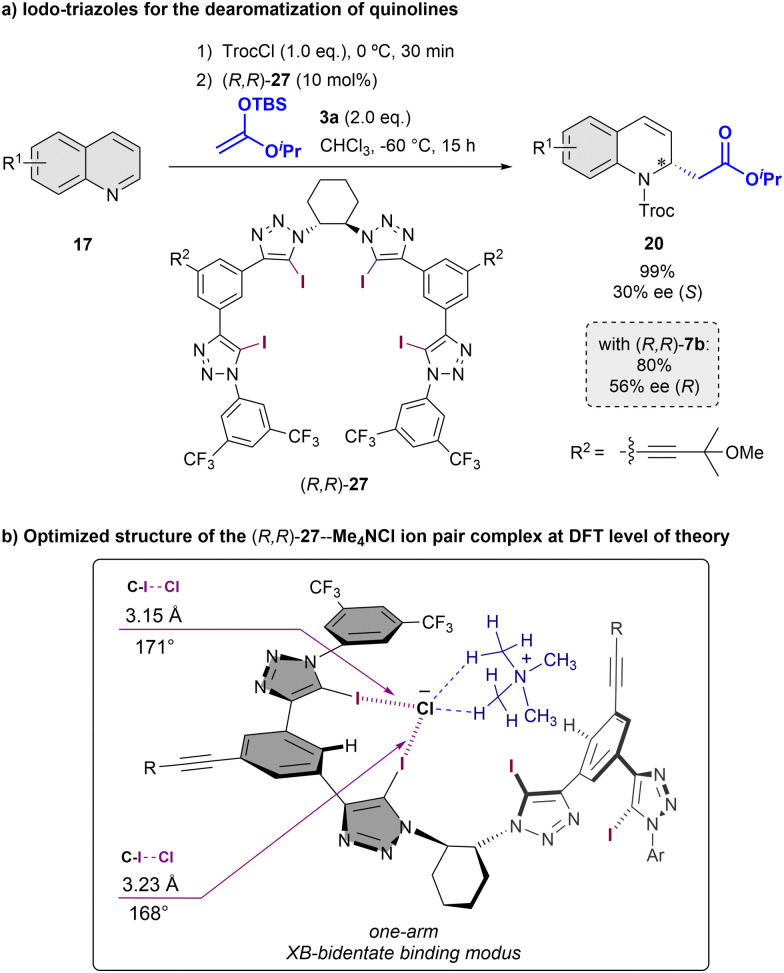
Asymmetric Reissert type reaction of quinolines employing a chiral triazole-based XB-donor catalyst (a) and computed binding mode with Me_3_NCl as model substrate (b).

Inspired by our previous results with chiral-triazole based anion-binders, in 2021 we reported the chiral tetrakis-iodo-triazole (*R*,*R*)-27 as a novel XB-donor organocatalyst (Scheme 8).^70^ To test its enantioinduction capability, the Reissert-type reaction of quinolines 17 with silyl ketene acetals 3 was chosen as a benchmark reaction. The anion binding properties of (*R*,*R*)-27 were examined by NMR- and CD-titrations using the corresponding tetrabutylammonium salt, showing significant stronger anion affinities compared to the analogous HB-donor (*R*,*R*)-7b (K_a_(Cl^−^) = 1125 *vs.* 99 M^−1^ in 1 : 1 acetone-d_6_ : CDCl_3_, 2.5 mM; Bu_4_NCl as Cl^−^ source). The best result was observed using 10 mol% of (*R*,*R*)-27 in CHCl_3_ at −60 °C. The desired (*S*)-product was the obtained in 30% ee; at the same level of the current state-of-the-art stereocontrol in XB-activation catalysis. Interestingly, the opposite enantiomer respect to the obtained with the parent proto-triazole catalyst (*R*,*R*)-7b was observed, which was in concordance to the examined pseudo-inverted CD-spectra and DFT calculations. These studies indicate that the bigger size of the iodine atoms in the upper triazoles produces a distortion of the helical cavity, leading to a bidentate binding mode instead of the tetradentate exhibited in the case of 7.

## 4. Dearomatization of pyridines

Pyridines, the simplest six-membered-ring N-heteroarenes, are very interesting molecules because present a broad bioactive spectrum.^71–73^ The dearomatization of pyridines is more challenging compared to the quinolines or isoquinolines. This could be attributed to two main issues. In the one hand, the complete loss of aromaticity in the case of pyridines, whereas quinolines or isoquinolines are able to partially retained it in the other aromatic ring. On the other hand, pyridines show additional difficulties in terms of regioselectivity due to the two possible positions, C2 and C4, prone to nucleophilic attack.^74^

**Scheme 9:**
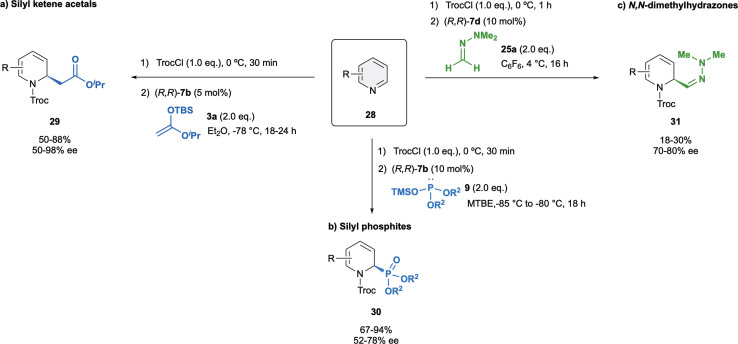
Dearomatization of pyridines with several nucleophiles reported by García Mancheño *et al*.

Challenged by their intrinsic reactivity and regioselectivity issues, in 2015 we developed the first enantioselective dearomatization of pyridines 28 mediated by anion binding catalysis (Scheme 9a).^75^ We were delighted to observed that (*R*,*R*)-7b was superior to other classical catalysts such as thiourea or squaramides regarding to both stereo- and regioselectivity. The reaction was carried out with 5 mol% of (*R*,*R*)-7b and iso-propyl ketene acetal 3a in Et_2_O at −78 °C, providing selectively the corresponding C2-substituted (*R*)-dihydropyridines 29 in high yields and up to 98% enantioselectivity. Moreover, the opposite (*S*)-enantiomer could also be obtained in a high enantiomeric ratio (−92% ee) when using the enantiomeric catalyst (*S*,*S*)-7b. It is important to remark that the C4 addition was preferred only with pyridines substituted at the C3 with medium or bulky groups or in absence of catalyst.

Our group also exemplary demonstrated the asymmetric dearomatization of pyridines with different silyl phosphites 9 with (*R*,*R*)-7b in MTBE as a solvent (Scheme 9b),^48^ as well as *N*,*N*-dimethylhydrazone 25a with catalyst (*R*,*R*)-7d in C_6_F_6_ (Scheme 9c).^52^ The desired products 30 and 31 were obtained in good to moderate yields, and enantioselectivities up to 78% ee and up to 80% ee, respectively.

**Scheme 10:**
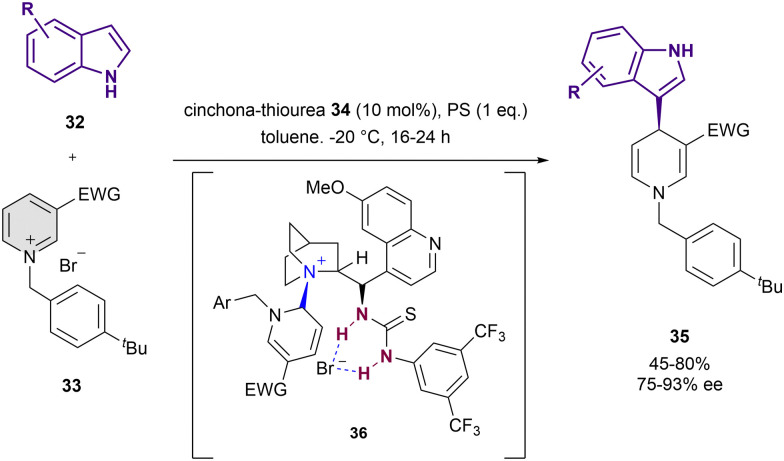
Asymmetric dearomatization of *N*-benzylpyridinium salts with indoles by Bernardi *et al*.

Thiourea-based catalysts have been employed for the dearomatization of pyridines too. In 2016, Caruana, Fochi and Bernardi reported the dearomatization of *N*-alkylpyridinium salts 33 instead of the more reactive acyl analogues by nucleophilic addition of indoles 32 (Scheme 10).^76^ The reaction was performed in presence of 10 mol% of 34 and stoichiometric amounts of 1,8-bis(dimethylamino)naphthalene that acts as a proton sponge (PS) in toluene as a solvent. The resulting 1,4-dihydropyridine derivatives 35 were obtained in high yields and up to 93% ee. The formation of the adduct 36 in the course of the reaction was observed by NMR. In addition to the expected interaction with the bromide counter-anion, it shows a covalent bond between the C6 atom of the pyridinium salt and the tertiary amine of 34. The simultaneous anion-binding activation and C2-blocking by covalent-bond formation with the catalyst explain the observed selective nucleophilic addition to the C4 position, resembling the normal reactivity of pyridinium salts.

## 5. Dearomatization of other N-heteroarenes

Besides isoquinolines, quinolines and pyridines, the anion-binding dearomatization approach has been also extended to more complicated structures which present more than one heteroatom such as diazarenes.^7,77^ These derivatives present a larger number of reactive sites, making a challenge to achieve not only stereo-, but also regiocontrol in the formation of the corresponding chiral di- or tetrahydrodiazaheterocycles.

**Scheme 11:**
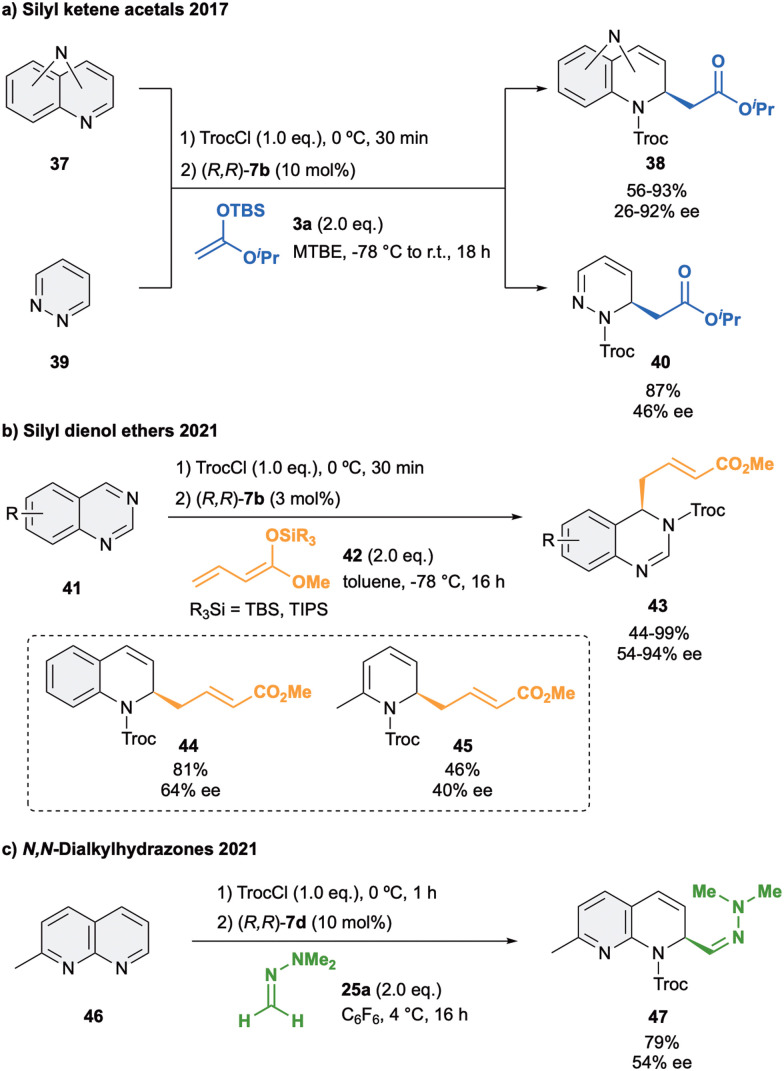
Enantioselective dearomatization of diazarenes with (a) silyl kentene acetals, (b) silyl dienol ethers and (c) dialkylhydrazones reported by García Mancheño *et al*.

In 2016, our group reported the first anion binding dearomatization reaction of various types of diazarenes with silyl ketene acetal 3a in presence of 10 mol% of (*R*,*R*)-7b in MTBE (Scheme 11a).^78^ The studied substrates 37 contained the two nitrogen atoms either in the same or in different rings, leading to the desired products 38 in high yields and enantioselectivities up to 92% ee. Moreover, more challenging six- and five-membered monocyclic diazarenes were also explored. In the case of the six membered heterocycle 39, the desired C2 product 40 was obtained with high regioselectivity, whereas the five membered heterocycles mostly led to decomposition products.

**Fig. 3:**
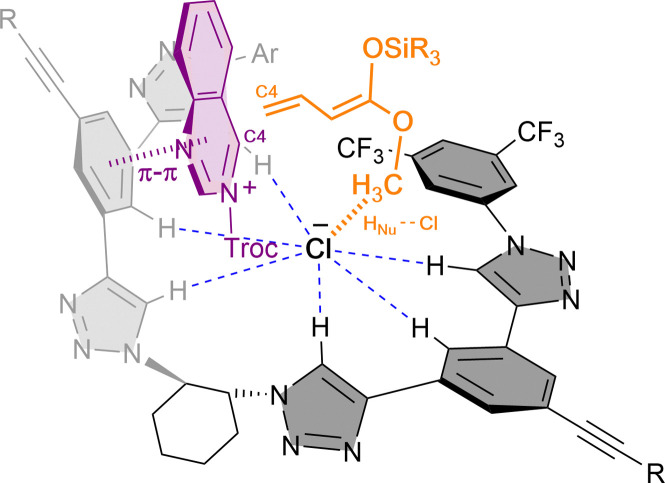
Catalyst-substrate-nucleophile complex *via* multiple non-covalent interactions proposed for the vinylogous Mukaiyama-type reaction reported by García Mancheño *et al*.

In 2021, we developed the vinylogous Mukaiyama-type dearomatization reaction of *N*-Troc activated quinazolines 41 by addition of silyl protected dienol ethers 42 in toluene (Scheme 11b).^79^ The use of 3 mol% of (*R*,*R*)-7b allowed for high selectivity, leading to the exclusive formation of the C4 addition products 43 in moderate to high yields and enantioselectivities up to 94% ee. A similar contact ion pair complex formation involving the fixation of the cationic substrate by π–π stacking with one of the arms of the catalyst and coulomb interactions with the chloride anion was proposed (Fig. 3). However, in this case, the additional interactions between the counterion and the dienolate (H_Nu_–Cl) orientate its vinylogous nucleophilic site towards the addition at the C4 position of the quinazoline from the less hindered Re-face. Interestingly, a less efficient orientation and stereocontrol was observed for other N-heteroarenes such as quinolines (44, up to 64% ee) or pyridines (45, up to 40% ee).

Lastly, an isolated example on the addition of *N*,*N*-dimethylhydrazone 25a to 2-methyl-1,8-naphthyridine (46) in presence of 10 mol% of (*R*,*R*)-7d was reported (Scheme 11c).^52^ The corresponding hydrazone 47 was then obtained in 79% of yield and a moderate 54% ee, showing again the importance of appropriate matching of the substrate, nucleophile and structural features of the used catalyst.

## 6. O-Heteroarenes: pyrylium-type derivatives

O-Heteroarenes, similarly to N-heteroarenes, are important synthetic building blocks with a rich chemistry towards the preparation of interesting naturally occurring and bioactive oxygen heterocyclic compounds.^80–82^ In this regard, pyrylium derivatives are well-suited substrates to test the anion-binding catalytic dearomatization approach. Nevertheless, some complications regarding their reactivity exists, because most of nucleophilic reactions with pyrylium salts lead to ring opening or decomposition reactions.^83^

### 6.1. Cycloaddition strategies

**Scheme 12:**
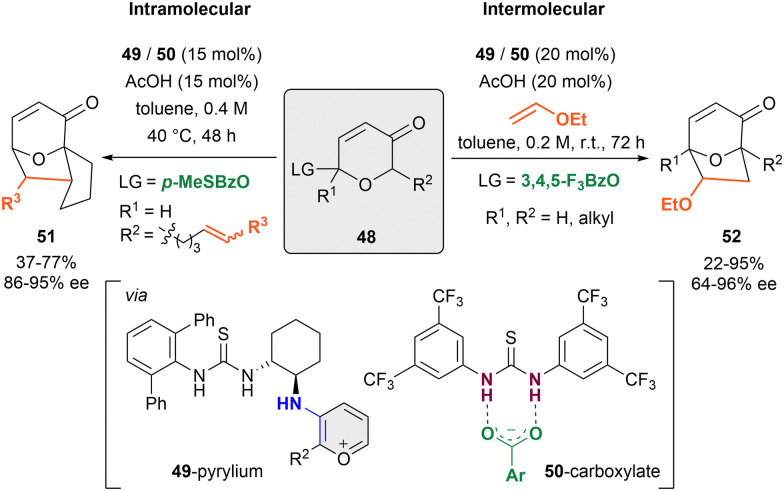
[5+2] cycloaddition of pyrylium derivates reported by Jacobsen *et al*.

In 2011, the Jacobsen group published the first anion-binding dearomatization reaction of O-heteroarenes by an intramolecular [5+2]-cycloaddition of 3-pyranones 48 bearing a carboxyl leaving group (LG) in the C2-position and a tethered alkene in C6 (*R*^2^) (Scheme 12, left).^84^ This method enabled the preparation of 8-oxabicyclo[3.2.1]octane derivatives 51 with α,β-unsaturated units. A dual cooperative thiourea organocatalytic system formed by the chiral primary amino-thiourea 49 (15 mol%), and an achiral thiourea 50 (15 mol%) was employed with acetic acid as a cocatalyst in toluene. First, the condensation between 49 and the ketone group of the substrate takes place to generate the corresponding reactive oxidopyrylium species upon enamine formation, followed by the benzoate-LG abstraction by 50 towards the assembling of the chiral contact ion pair between the 49-pyrylium and 50-carboxylate complexes. The desired cycloadducts 51 were obtained in moderate yields and enantioselectivities up to 95% ee.

Later on, the same group was able to achieve higher yields and enantioselectivities through a related intermolecular [5+2]-cycloaddition between substituted pyrylium derivatives and electron rich terminal alkenes such as vinylethers (Scheme 12, right).^85^ However, a slightly higher catalytic loading (20 mol% *vs.* 15 mol%) and the use of 3,4,5-trifluorobenzoate (3,4,5-F_3_BzO) as leaving group were required for an optimal stereochemical outcome in the products 52 (up to 96% ee).

### 6.2. Nucleophilic additions

**Scheme 13:**
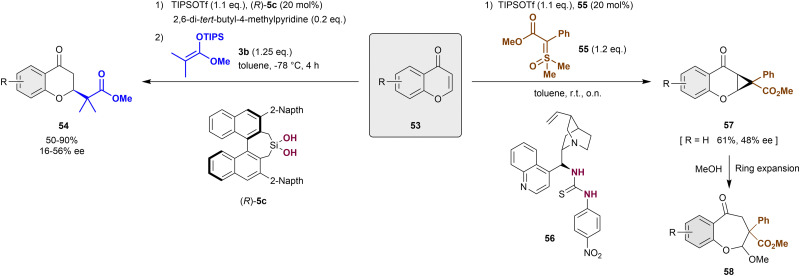
Enantioselective dearomatization of *in situ* formed benzylpyrylium derivatives reported by Mattson.

Alternative to the dearomatization of pyrylium derivatives by cycloaddition approaches, nucleophilic dearomatizations reactions have been also employed for this type of heteroarenes.^86,87^ In 2016, Mattson *et al.* described the first anion-binding catalytic addition of carbonyl nucleophiles such as silyl ketene acetals 3 to chromanones 53 (Scheme 13, left).^86^ These substrates are previously activated *in situ* with TIPSOTf to form the reactive siloxybenzopyrylium salt substrates. The development of a new 3,3′-naphtalen-2-yl silanediol (*R*)-5c was necessary to achieve the desired products 54 in high yields and enantioselectivities up to 56% ee. Compared to the previously used silanediols (see Scheme 2), (*R*)-5c presents a bulkier environment around the active binding site, which allows the formation of a unique chiral pocket.

More recently, the Mattson group reported the racemic cyclopropanation-nucleophilic ring expansion sequence towards the synthesis of benzo[*b*]oxepines 58 (Scheme 13, right).^87^ To achieve this, sulfoxonium ylides 55 were employed for the cyclopropanation of benzylpyrylium triflates, which were prepared *in situ* from chromanones 53 through the classical approach depicted above. Although the method was mainly developed as racemic version, the use of the chiral thiourea-based anion-binding catalyst 56 allowed a facially selective cycloaddition reaction. Thus, the corresponding model product 57 (R = H) was achieved in 61% yield and 48% ee, which was improved to 74% ee after recrystallization.

**Scheme 14:**
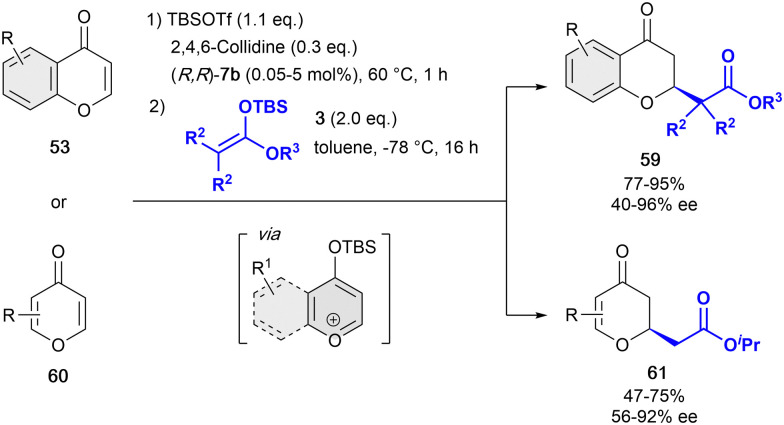
Dearomatization of pyrylium derivatives by García Mancheño *et al*.

In 2019, and inspired by the previous work of Mattson,^86^ we developed the one-pot dearomatization reaction of pyrylium-type derivatives with differently substituted silyl ketene acetals 3 in presence the tetrakistriazole (*R*,*R*)-7b (Scheme 14).^88^ This catalytic system showed a high reactivity, allowing for extremely low loadings (typically 1 mol%, and down to 0.05 mol%)^89^ compared to the previous reported studies, while maintaining a high enantiocontrol. The active benzylpyrylium salts were formed *in situ* by treatment of 4-chromanones 53 with 2,6-lutidine and *tert*-butyldimethylsilyl trifluoromethanesulfonate (TBSOTf) in toluene. Differently substituted OTBS-benzylpyrylium salts formed *in situ* from 4-chromones were well tolerated providing the corresponding products 59 with high yields and enantioselectivities up to 96% ee. Remarkably, this approach was successfully extended to the more challenging 4-pyrones 60 in presence of 5 mol% of (*R*,*R*)-7b, leading to no ring opening but the desired products 61 in moderate yields and enantioselectivities up to 92% ee.

## 7. Heteroarenes as nucleophiles in anion-binding dearomatization reactions

**Scheme 15:**
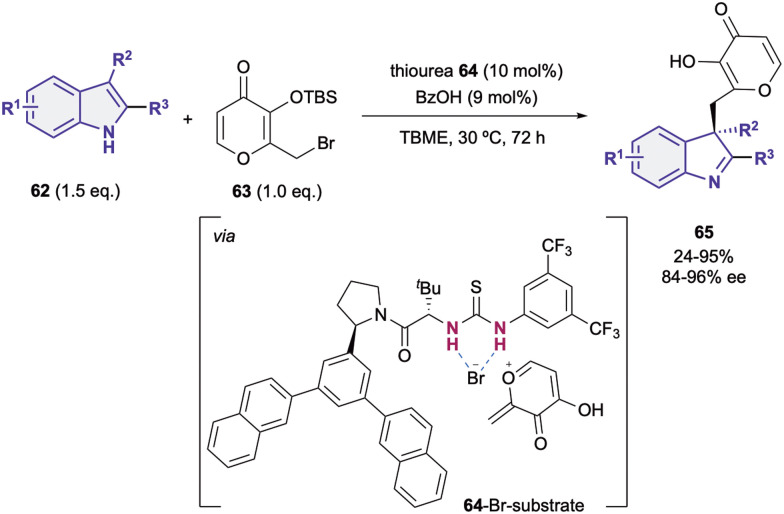
Indole addition to pyrones by Jacobsen and co-workers.

Although most of the dearomatization strategies based on anion-binding catalysis are based on the activation of the heteroarene as electrophile, electron rich heteroarenes can also be dearomatized through this type of catalysis upon reacting with the appropriate electrophilic species.^19,90,91^ Embracing this idea, in 2014, Porco Jr., Jacobsen *et al.* reported the nucleophilic addition of 3-substituted indoles 62 to silyl protected γ-pyrone derivatives 63 (Scheme 15).^90^ They employed an arylpyrrolidino-derived thiourea organocatalyst 64 in combination with benzoic acid as a cocatalyst. The desilylation of the pro-electrophile 63 promoted by the Brønsted acid^92^ takes first place, followed by the bromide-anion abstraction by 64 towards the formation of the chiral contact ion pair (64-Br-substrate). Finally, after the nucleophilic addition of the indole 62 to the *in situ* generated *exo*-conjugated oxonium electrophile, the desired chiral indoline products 65 bearing a quaternary stereocenter in the C3-position were obtained in moderate to high yields and enantioselectivities up to 96% ee.

**Scheme 16:**
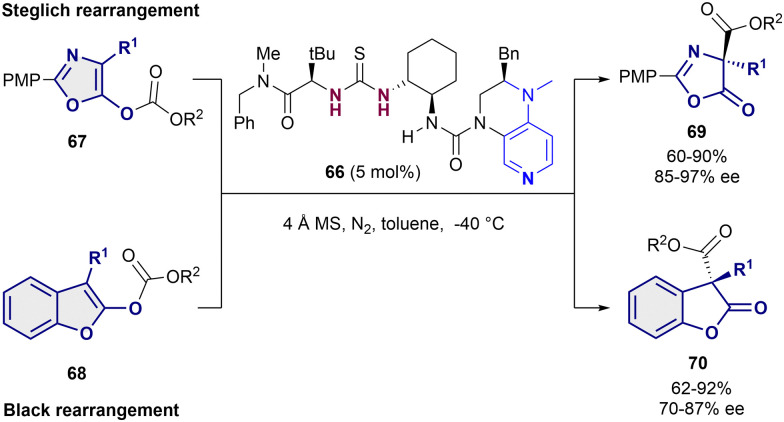
Enantioselective Steglich and Black rearrangements reported by Liu and Tian.

As last example, Liu and Tian employed the bifunctional thiourea-pyridine organocatalyst 66 for the acyl transfer in both a Steglich rearrangement of oxazolyl carbonates 67 and the Black rearrangement of benzofurane substrates 68 (Scheme 16).^91^ This bifunctional catalyst behaves in a similar way as the one described by Seidel in 2011,^46^ in which the dialkylaminopyridine moiety activates the acyl group and the thiourea behaves as a chiral anion receptor. In both cases, 5 mol% of 66 was enough to obtain moderate to high yields and enantioselectivities up to 97% ee for the Steglich reaction (69), and 87% ee for the Black rearrangement (70).

## 8. Conclusions & outlook

Anion binding organocatalytic approaches have proved to be highly efficient to achieve the dearomatization of heteroarenes. After the pioneering studies in this field with chiral thioureas by the group of Jacobsen, several other catalytic systems such as silanediols, oligo-triazoles and bifunctional catalysts have been introduced for the dearomatization of electron poor N-hetereoarenes such as (iso)quinolines, pyridines and diazarenes using silyl ketene acetals as nucleophiles. Additionally, other nucleophiles such as silyl phosphites, allyltin reagents, dienolates, or *N*,*N*-dialkylhydrazones were also successfully applied. Furthermore, this approach was efficiently extended to highly challenging pyrylium-type O-heterocycles, which was also possible through a pre-activation of non-aromatic chromanone or pyrone derivatives by enamine formation or enolization followed by anion-binding catalysis. Alternative to the most employed activation and dearomatization of electrophilic heteroarenes, a few examples with electron rich aromatic compounds have been also described. In these cases, the heteroarene plays the role of the nucleophile, whereas the anion-binding catalyst activates the electrophilic reaction partner. Finally, inspired by classical HB-catalysis, the first application of XB-activation was also employed for this approach, opening new possibilities for the future design of catalysts relying in σ-hole interactions. However, despite some important breakthroughs in the field covered here, the design of more effective anion-binding catalysts for enantioselective dearomatization reactions combined with a low catalyst loading is still a real challenge due to the fact that ion pairing is less directional than covalent interactions.

The results discussed in this feature provides a solid platform and shows broad possibilities for the future exploration of the chemical landscape within this research field, where we anticipate that dual and bifunctional synergetic catalytic systems will have an important impact in the design of more efficient catalytic dearomatization processes beyond the to date addressed heteroarenes and the discovery of new catalytic transformations.

## Author contributions

Both authors have discussed the organization and presentation of the contents, and participated on the writing and editing of the Feature Article.

## Conflicts of interest

There are no conflicts to declare.

## Supplementary Material
